# Quantitative evaluation of retinal microvasculature and retrobulbar vessels after intravenous chemotherapy for retinoblastoma

**DOI:** 10.1186/s12886-021-02170-4

**Published:** 2021-11-26

**Authors:** Xiuqian Yi, Jie Sun, Jiang Qian, Jie Guo, Kang Xue

**Affiliations:** 1 Department of Ophthalmology, Eye, Ear, Nose, and Throat Hospital of Fudan University, Shanghai, 200031 China; 2 Key Laboratory of Myopia of the State Health Ministry, and Key Laboratory of Visual Impairment and Restoration of Shanghai, China, Shanghai, China

**Keywords:** Retinoblastoma, Retrobulbar ocular blood flow, Optical coherence tomography angiography, Intravenous chemotherapy

## Abstract

**Backgroud:**

To evaluate the changes in retinal microvasculature and retrobulbar blood flow, using optical coherence tomography angiography (OCTA) and Color Doppler imaging (CDI) after intravenous chemotherapy (IVC) in patients with retinoblastoma (RB).

**Methods:**

This was a retrospective comparative case control series involving 30 patients. Ten bilateral RB patients that had a preserved eye with extramacular tumours (group I), 10 unilateral RB treated with IVC that had a normal fellow study eye (group II), and 10 age-matched healthy controls. The macular retinal thickness, foveal avascular zone (FAZ) area, and the macular and peripapillary retinal vessel densities (RVD) were measured. The peak systolic and end diastolic velocities of the ophthalmic, central retinal and posterior ciliary arteries were determined. A comparison among the three groups was conducted.

**Results:**

Between the three cohorts, OCTA revealed no significant difference in FAZ area, superficial foveal and parafoveal RVD, deep parafoveal RVD and peripapillary RVD, (*P* > 0.05). By contrast, the mean deep foveal RVD, the full, inner and outer foveal and the parafoveal retinal thickness were significantly lower in group I compared with the controls, (*P* = 0.0329, 0.0153, 0.0311 0.0352, 0.0215). No significant difference in the blood flow velocities occurred in the retrobulbar circulation (*P* > 0.05).

**Conclusions:**

In patients with retinoblastoma, OCTA did not detect significant changes of retinal thickness and vessel density in the eyes treated with IVC, but a slight reduction in retinal thickness and the deep foveal RVD seemed to occur in bilateral RB eyes. The retrobulbar blood flow parameters showed no measurable changes.

## Backgroud

Retinoblastoma (RB) is the most common, primary, intraocular malignancy in children. The improvement in globe salvage is mostly related to the introduction of intravenous chemotherapy (IVC, chemoreduction) coupled with focal consolidation as the most commonly used strategy for RB worldwide [1, 2]. In recent years, advances in treatment options, such as highly selective intra-arterial have reduced the use of systemic chemotherapy [3]. Although there can be systemic toxicities associated with IVC, such as bone marrow suppression and hearing loss [4, 5], limited studies have reported on ophthalmic changes after IVC using spectral domain optical coherence tomography (OCT) [6] and optical coherence tomography angiography (OCTA) [7].

OCTA is a novel, non-invasive method and its recent use, along with the split-spectrum amplitude-decorrelation angiography (SSADA) algorithm, permits the visualisation of the vascular system of the retina at the capillary level. It has also been used to study the microvasculature effects of benign and malignant intraocular tumours [8–10].

In the previous study, we demonstrated that intra-arterial chemotherapy (IAC) had a measurable effect on the retrobulbar blood flow [11]. However, there are limited reports in the literature about CDI changes of these vessels after IVC. In this study, we evaluated the effects of IVC on the retrobulbar and ocular blood flow parameters in patients with retinoblastoma via CDI and OCTA, to understand the underlying ocular toxicities in the paediatric population.

## Methods

### Ethics approval and consent to participate

The Institutional Review Board at Eye, Ear, Nose, and Throat Hospital of Fudan University approved this study. Written informed consent was obtained from the parents, caretakers, or guardians on behalf of all of the children.

### Patients

The retrospective study was conducted at the Department of Ophthalmology, Eye, Ear, Nose and Throat Hospital of Fudan University in Shanghai, China, from January to December 2020. Consecutive patients with RB treated with IVC and enucleation performed earlier in life and currently between the ages of 7 and 15 years were enrolled in this study. The authors divided the eyes into three groups: bilateral RB patients that had a preserved eye with extramacular tumours, with the staging as Group A-C at diagnosis (group I, *n* = 10), unilateral RB treated with IVC that had a normal fellow study eye (group II, *n* = 10), and age-matched healthy controls (control group, *n* = 10).

Subjects with any of the following were excluded: newly diagnosed RB cases and those with less than 1 year follow-up time following the complete stoppage of systemic chemotherapy and/or local therapy; patients who had received external beam radiotherapy (brachytherapy) and subconjunctival chemotherapy, intraarterial or intravitreal chemotherapy; with tumours affecting the macular and optic disc region (edge of tumour was less than 3 mm from fovea and optic disc boundary); or with macular subretinal fluid, cystoid macular edema, vitreous haemorrhage or cataract.

### Ophthalmological examination

All the children had a full history taken which included their age at the time of the present study, sex, age at the time of first presentation of RB, duration of follow-up, family history, modality of treatment used, number of IVC cycles, international classification of retinoblastoma (ICRB), number of tumours, distance to optic disc (mm) and to foveal (mm). All patients underwent comprehensive ophthalmologic examinations, which included best-corrected visual acuity (BCVA), refraction measurement using auto refraction, spherical equivalence (SE) calculation, slit-lamp biomicroscopy, dilated fundus examination and axial length (AL) measured with an optical biometry device (IOLMaster; Carl Zeiss AG, Jena, Germany).

### Optical coherence tomography angiography

OCTA was employed using a spectral domain system RTVue-XR Avanti (Optovue, Fremont, CA, USA). This system has an A-scan rate of 70 kHz per second with a light source centred at a wavelength of 840 nm and a bandwidth of 45 nm [12–14]. Two volumetric raster scans, including one horizontal priority (x-fast) and one vertical priority (y-fast), were obtained consecutively for each area and repeated again. The SSADA algorithm was employed to improve the signal-to-noise ratio by splitting the spectrum to generate multiple repeat OCT frames from the two original repeat OCT frames [15], and any motion artefacts were removed with 3-D orthogonal registration and the merging of the two scans.

Macular (6 × 6 mm) and optic disc (4.5 × 4.5 mm) OCT angiography scans were acquired. The vessel densities in the foveal, parafoveal and peripapillary areas were assessed as follows. The foveal area was a 1 mm diameter circle centred on the fovea. The parafoveal area was defined as an annulus with an outer diameter of 3 mm and an inner diameter of 1 mm centred on the fovea. The peripapillary area was defined as a 700 μm wide elliptical annulus extending outward from the optic disc boundary. The retinal vessel densities of the specific areas were defined as the percentage area occupied by vessels in the corresponding segmented areas [13].

At the same time, the FAZ area and macular thicknesses were measured using the same OCTA system. The FAZ was automatically outlined and measured by built-in ImageJ software. The macular thicknesses, including the foveal and parafoveal area, were obtained using the retinal map protocol. The full retinal thickness was measured from the internal limiting membrane to the middle of the retinal pigment epithelium and the inner retinal thickness from the internal limiting membrane to the outer boundary of the inner plexiform layer. The retinal thicknesses of each area were automatically determined using the system’s software and were defined as the mean thicknesses of each area.

### Colour Doppler imaging measurements

The HDI 5000 CDI (Philips Ultrasound, Bothell, Washington, USA) with a 7.5 MHz linear probe was used to evaluate the blood flow of retrobulbar vessels posterior ciliary artery (PCA), central retinal artery (CRA) and ophthalmic artery (OA)). The patients were placed in the supine position and examined by the same experienced sonographer. All processes were done in accordance with the CDI measurements protocol, as described previously [16]. The peak systolic velocity (PSV) and end diastolic velocity (EDV) in each vessel were measured. The pulsatility (PI) and resistance (RI) indices were calculated automatically by the scanner.

### Statistical analyses

For the analyses of the data, the Statistical Package for the Social Sciences version 17.0 (SPSS, Chicago, IL) was used, and the descriptive statistics included the standard deviations of the means for all of the variables. The categorical variables were compared using unpaired S tudent ‘s t-test, χ or Fischer’s tests, and the continuous variables between the groups were compared using the Mann–Whitney U and Kruskal–Wallis tests. *P* < 0.05 was considered statistically significant.

## Results

### Basic ophthalmic assessment

Overall, 30 patients (30 eyes) of three groups were included, with a mean age of 9.4 ± 2.2 years (range, 7–15 years). The demographic and clinical characteristics of the RB patients and healthy controls are summarised in Table 1. There was no significant difference between the three groups concerning their age, visual acuity and axial length (*P* > 0.05). In addition, the duration from last IVC to time of the exam and the number of IVC cycles were similar between group I and II (*P >* 0.05). The age at the time of the first presentation of RB was significantly younger in group I (*P <* 0.05).

**Table 1:** The demographic and clinical data of the patients with RB and healthy controls

	Group I	Group II	Control	*P* value
Number of subjects	10	10	10	
Age (years)	9.7 ± 2.4	8.8 ± 2.3	9.6 ± 1.7	
Gender, n				
Male	5	4	6	
Female	5	6	4	
Tumor consolidation, n				
Cryotherapy	8	0	0	
TTT	9	0	0	
Cryotherapy + TTT	7	0	0	
BCVA (logMAR)	0	0	0	
Axial Length (mm)	23.0 ± 1.1	22.9 ± 0.8	23.1 ± 0.7	
First presentation time (mouth) (mouth)	12.9 ± 7.7	30.1 ± 9.6		**0.0003**
Duration time (year)	8.2 ± 2.4	7.3 ± 2.4		0.4127
IVC times	6.5 ± 1.6	6.5 ± 1.6		1
Family history, n (%)	1(10%)	0		

Values are expressed as the mean ± SD. *p* < 0.05 was considered statistically significant

*BCVA* Best-corrected visual acuity, *logMAR* Logarithm of the minimum angle of resolution, *IVC* Intravenous chemotherapy. *Duration time* Duration from last IVC to time of the exam

### OCTA parameters, retinal thickness and retinal vessel densities (RVD)

**Fig. 1:**
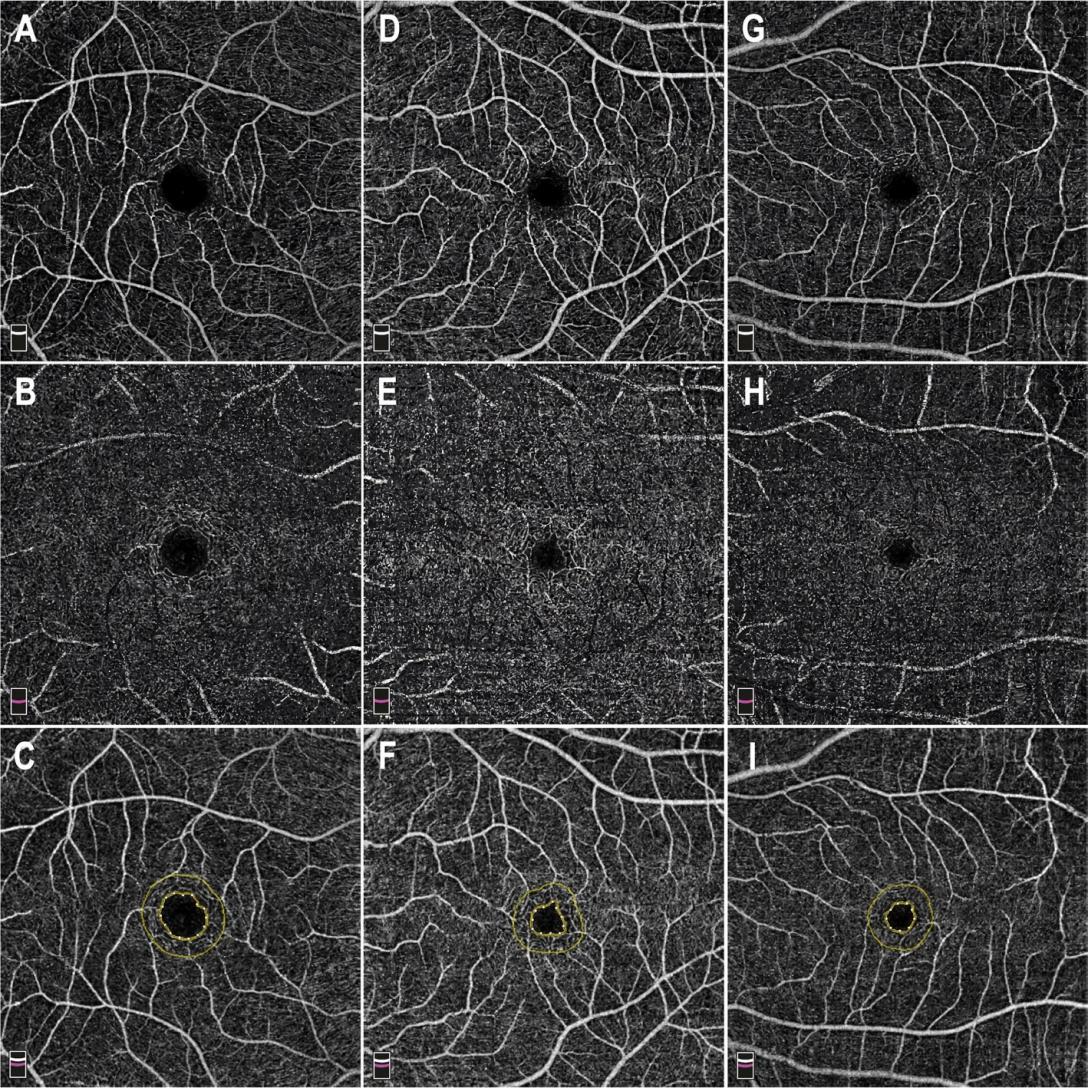
The example of optical coherence tomography angiography (OCTA) image in three groups. En face image (6 × 6 mm) of the corresponding layer of the superficial (**A**) and deep (**B**) capillary plexuses, and the area of the foveal avascular zone (FAZ) (**C**) was automatically calculated in Group I (0.445) (right eye, bilateral RB had a preserved eye with extramacular tumors). En face of the corresponding layer of the superficial (**D**) and deep (**E**) capillary plexuses, and the area of the foveal avascular zone (FAZ) (**F**) in Group II (0.224) (left eye, unilateral RB with a normal eye). En face of the corresponding layer of the superficial (**G**) and deep (**H**) capillary plexuses, and the area of the foveal avascular zone (FAZ) (I) in control Group (0.203) (left eye). Capillary density was calculated as percent of vessels per en face image for both plexuses. The mean superficial plexus capillary density did not demonstrate statistically significant difference in three groups. By contrast, mean deep plexus capillary density was significantly reduced in group I compared with controls

**Fig. 2:**
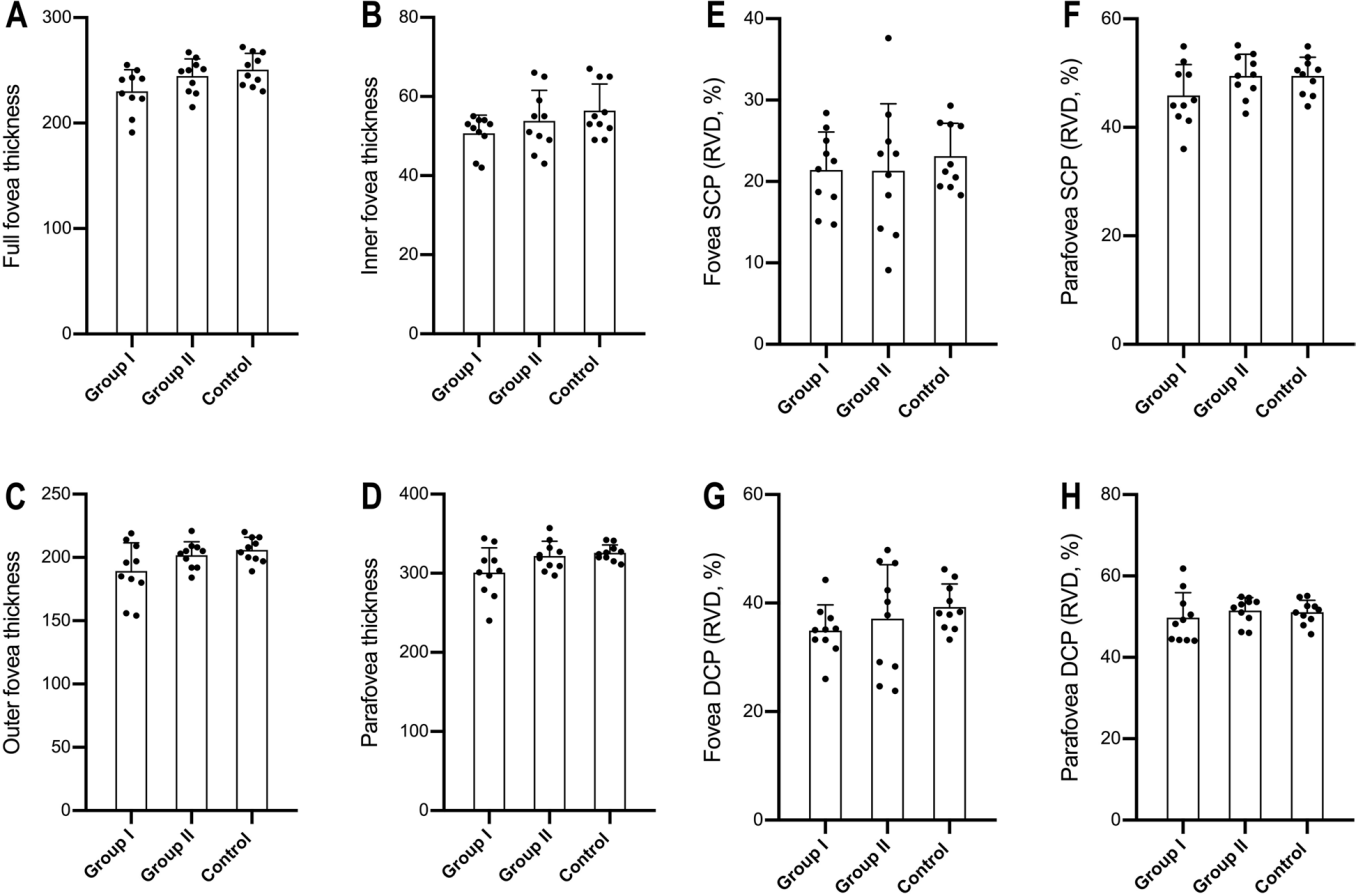
A comparison among the three groups was conducted, including the macular retinal thickness (**a** full foveal, **b** inner foveal, **c** outer foveal, **d** parafoveal), foveal avascular zone (FAZ) area, and the macular and peripapillary retinal vessel densities. As the retinal vessel densities (RVD) in superficial foveal (**e**) and parafoveal (**f**), the deep foveal (**g**) and deep parafoveal (**h**). By contrast, the mean deep foveal RVD, the full, inner and outer foveal and the parafoveal retinal thickness were significantly lower in group I compared with the controls, (*P* = 0.0329, 0.0153, 0.0311 0.0352, 0.0215)

Comparing group I with the control group, the retinal vessel densities in the deep foveal was significantly lower in group I (34.9% vs 39.3%, *P* = 0.0329). The full, inner and outer foveal and parafoveal retinal thickness were significantly thinning in group I (230.0 vs 250.7, *P* = 0.0153; 50.7 vs 56.4, *P* = 0.0311; 189.3 vs 206.0, *P* = 0.0352; 300.7 vs 325.7, *P* = 0.0215). However, there was no significant difference in the FAZ area, the RVD in the superficial foveal and parafoveal area, the RVD in the deep parafoveal area and the RVD in the peripapillary area (all *P* > 0.05) (Table 2) (Figs. 1 and 2).

**Table 2:** Retinal Thicknesses and Vessel Densities of three groups

Group	I	II	Controls		*P* value	
				I vs II	I vs C	II vs C
Fovea thickness (μm)						
Full	230.0 ± 19.5	244.5 ± 15.5	250.7 ± 14.7	0.082	**0.0153**	0.3709
Inner	50.7 ± 4.3	53.8 ± 7.4	56.4 ± 6.4	0.2670	**0.0311**	0.4117
Outer	189.3 ± 21.2	201.8 ± 10.0	206.0 ± 9.4	0.1090	**0.0352**	0.3460
Parafoveal thickness (μm)	300.7 ± 29.9	321.8 ± 17.6	325.7 ± 9.6	0.0704	**0.0215**	0.5461
Choroidal thickness	266.4 ± 84.7	288.3 ± 84.1	312.6 ± 82.7	0.5690	0.2330	0.5230
FAZ area (mm2)	0.257 ± 0.088	0.247 ± 0.133	0.237 ± 0.058	0.8450	0.5559	0.8299
SCP (RVD, %)						
foveal	21.4 ± 4.4	21.3 ± 7.8	23.1 ± 3.8	0.9722	0.3674	0.5201
Parafoveal	45.9 ± 5.4	49.5 ± 3.8	49.5 ± 3.3	0.1018	0.0888	1.0000
DCP (RVD, %)						
foveal	34.9 ± 4.5	37.1 ± 9.4	39.3 ± 4.0	0.5129	**0.0329**	0.5045
Parafoveal	49.8 ± 5.8	51.5 ± 3.1	51.1 ± 2.8	0.4244	0.5313	0.7655
Peripapillary (RVD, %)	50.7 ± 3.5	53.3 ± 1.9	52.5 ± 2.2	0.0537	0.1854	0.2910
pRNFL	119 ± 32.1	124 ± 12.0	117 ± 12.4	0.6501	0.8562	0.2158

Values are expressed as the mean ± SD. *P* < 0.05 was considered statistically significant

*RVD* Retinal vessel density, *SCP* Capillary plexus, *DCP* Deep capillary plexus

Comparing group I with II, and group II with the control group respectively, there were no statistically significant differences in the FAZ area, the RVD in the superficial foveal and parafoveal area, the RVD in the deep foveal and parafoveal area and the RVD in the peripapillary area. The full, inner and outer foveal and parafoveal retinal thickness were similar (*P* > 0.05; Table 2).

### Retrobulbar blood flow parameters

The mean retrobulbar blood flow values of the OA, PCA, and CRA among the three groups are shown in Table 3. There were no statistically significant differences in all the parameters.

**Table 3:** Hemodynamic Doppler parameters in the OA, PCA, and CRA of Eyes After Intravenous Chemotherapy for RB

Parameter	Group I	Group II	Controls	*P* value		
				I vs II	I vs C	II vs C
Central retinal artery						
PSV (cm/s)	11.28 ± 3.52	11.52 ± 3.73	11.72 ± 2.92	0.8840	0.7644	0.8953
EDV (cm/s)	3.82 ± 1.42	3.52 ± 1.64	3.71 ± 1.84	0.6671	0.8827	0.8102
RI	0.66 ± 0.07	0.69 ± 0.08	0.68 ± 0.09	0.3839	0.5859	0.7958
PI	1.12 ± 0.23	1.18 ± 0.21	1.15 ± 0.21	0.5500	0.7642	0.7531
Posterior ciliary artery						
PSV (cm/s)	11.94 ± 3.08	12.21 ± 3.24	12.52 ± 3.43	0.8507	0.6954	0.8377
EDV (cm/s)	3.67 ± 1.18	3.84 ± 1.64	3.73 ± 1.48	0.7932	0.9213	0.8766
RI	0.69 ± 0.09	0.69 ± 0.10	0.70 ± 0.10	1.0000	0.8168	0.8256
PI	1.06 ± 0.23	1.04 ± 0.25	1.04 ± 0.22	0.8544	0.8447	1.0000
Ophthalmic artery						
PSV (cm/s)	29.40 ± 6.63	30.47 ± 5.63	31.47 ± 5.6	0.7018	0.4604	0.6951
EDV (cm/s)	6.82 ± 2.62	7.11 ± 2.34	6.98 ± 3.12	0.7970	0.9025	0.9172
RI	0.77 ± 0.08	0.76 ± 0.09	0.78 ± 0.10	0.7958	0.8078	0.6439
PI	1.25 ± 0.38	1.24 ± 0.40	1.27 ± 0.43	0.9549	0.9135	0.8735

*OA* Ophthalmic artery, *PCA* Posterior ciliary artery, *CRA* Central retinal artery, *PSV* Peak systolic velocity, *EDV* End diastolic velocity

## Discussion

Choroidal thinning and ischemic atrophy after the treatment of intraarterial chemotherapy in RB have been documented in vivo using EDI-OCT. [17] Our previous results suggested that some significant decreases in the blood flow velocities occurred in the retrobulbar circulation in some arteries after IAC [11]. Although IVC has been widely used in RB treatment for over 20 years, limited previous observations of ocular toxicities, including retinal ischemia after IVC, have been published. Shields et al [7] reported a slight reduction in capillary density of the deep capillary plexus in patients after IVC for RB without alterations in the central macular thickness or choroidal thickness. However, there are few reports in the literature about CDI changes of these vessels after IVC.

In this study, we documented that the deep foveal RVD were significantly lower in the preserved eye with extramacular tumours, while the full, inner and outer foveal and the parafoveal retinal thickness were also significantly decreased. Standard IVC for RB includes vincristine and etoposide, and carboplatin, and the related toxicities are minimal. CRA and PCA, branches of OA, supply blood to the inner retina and choroid layer, respectively, through which the chemotherapeutic drugs enter the eyeballs. In our study, there were no measurable changes in the blood flow velocities in the PCA, CRA or OA. These drugs have not yet been observed to cause direct ocular toxicities in RB patients. Perhaps chemotherapy-induced micro-ischemia is below the threshold of causing blood flow velocity changes and visual acuity changes. Instead, IAC may trigger vascular toxicity through endothelial cell inflammation and leukostasis, leading to ocular and orbital vessel toxicity [18, 19]. IVC continues to have a safe ocular toxicity profile and work as a major treatment for early stage retinoblastoma. The current indications for IVC include patients with bilateral RB, confirmed germline mutation, familial retinoblastoma, aged 4 months or younger and high-risk histopathologic features [20, 21].

In this study, eyes without tumours that received chemotherapy (because of the opposite eye with the tumour [group II]) showed no signs of microischemia in OCTA or blood flow velocity changes in CDFI compared with the normal control. While the bilateral RB patients that had a preserved eye with extramacular tumours showed significantly decreased deep foveal RVD and alterations in the full, inner and outer foveal and the parafoveal retinal thickness. We interpreted this finding to represent an effect of focal consolidation or a tumour effect rather than a chemotherapy effect. The focal consolidation, including cryotherapy and transpupillary thermotherapy (TTT), might result in retinal and choroidal thinning and atrophy. Even the focal consolidation was conducted in the extramacular region, it might still have a measurable effect on the deep foveal RVD, and the mild subclinical retinal ischemia was detected only by OCTA.

Azza et al. [6] proposed that retinoblastoma eyes, characterised by thinning of the central fovea, GCL, GCC compared with the control group, and the changes may be due to the genetic defects in RB. Our results proposed that the full, inner and outer foveal and parafoveal retinal thickness were significantly thinning in group I. Bilateral RB patients that had a preserved eye with extramacular tumours, which had genetic defects, and were consistent with the results of Azza. We also compared the vessel densities in the peripapillary area, resulting in no significant difference among the three groups, while RNFL thicknesses and peripapillary RVD were also similar in all three groups which has not been reported before. The vessels in the peripapillary area originate from two systems, the central retinal artery and the short posterior ciliary arteries. The chemotherapy might not have had a measurable vascular toxicity, since vessel densities and the RNFL remained unchanged when chemotherapeutic drugs entered the eyeballs.

This study was not without certain limitations. First of all, we must mention that the number of participants included was limited to reach a statistically significant difference. Second, since the difference remained slight between the RB tumour and the control groups, a larger study may be required to validate this outcome. Additionally, OCTA with analyses is an examination that we could not perform in the supine position as it needs a patient’s cooperation. So far, therefore, it has not been possible to perform OCTA on patients under anaesthesia and on young infants, and the retinal thickness and vessel density before IVC treatment could not be detected. Finally, as IAC is more popular in the treatment of advanced Rb which seem to have more ocular toxicities, further study will be investigated on retinal microvasculature after IAC.

## Conclusion

In summary, our results suggested that in retinoblastoma patients, OCTA did not detect significant changes of the retinal thickness or vessel density in eyes treated with IVC, but a slight reduction in the foveal and parafoveal retinal thickness, and deep foveal RVD seemed to occur in bilateral RB eyes. These changes might be due to the effect of focal consolidation or the genetic defects in RB. No measurable changes in the blood flow velocities occurred in the retrobulbar circulation after IVC with a long-term follow-up.

## Data Availability

All of our detailed information were collected from doctor’s workstation of Eye and ENT Hospital of Fudan University, which is read-only, so we are sorry that the detailed data can’t be shared.

## Abbreviations

OCTA: Optical coherence tomography angiography
CDI: Color Doppler imaging
IVC: Intravenous chemotherapy
RB: Retinoblastoma
FAZ: Foveal avascular zone
RVD: Retinal vessel densities
OCT: Optical coherence tomography
SSADA: Split-spectrum amplitude-decorrelation angiography
IAC: Intra-arterial chemotherapy
BCVA: Best-corrected visual acuity
SE: Spherical equivalence
AL: Axial length
PCA: Posterior ciliary artery
CRA: Central retinal artery
OA: Ophthalmic artery
PSV: Peak systolic velocity
EDV: End diastolic velocity
PI: Pulsatility indices
RI: Resistance indices
